# Hemangioma of the umbilical cord: A case report and proposal for standardised reporting criteria

**DOI:** 10.1016/j.crwh.2025.e00708

**Published:** 2025-04-05

**Authors:** Isabella Charlotte Maréchal-Ross, Sashi Siva, Karen Mizia, Jeremy Nicholas Pulvers, Isabella Turton, Ali Moghimi

**Affiliations:** a Department of Obstetrics & Gynaecology, Royal North Shore Hospital, Clinical Services Building, 1 Westbourne St, St Leonards 2065, NSW, Australia; b Department of Anatomical Pathology, NSW Health Pathology, Gosford Hospital, 75 Holden St, Gosford, NSW 2250, Australia; c Department of Histopathology, The Children's Hospital at Westmead, Corner Hawkesbury Road and Hainsworth Street, Westmead, NSW 2145, Australia.

**Keywords:** Umbilical cord hemangioma, Umbilical cord tumour, Placental pathology, Vascular anomaly, Obstetric ultrasound, Perinatal management

## Abstract

Umbilical cord hemangiomas are rare benign vascular anomalies with limited documentation in the literature. Given their association with adverse perinatal outcomes, standardised criteria for reporting and monitoring are needed. This case report presents an instance of umbilical cord hemangioma and proposes a structured framework for future documentation.

A comprehensive literature review using OVID Medline and Embase identified cases of umbilical cord hemangiomas, their clinical presentations, and maternal and neonatal outcomes. A case diagnosed in the third trimester is presented, detailing antenatal surveillance, histopathological findings, and perinatal outcomes. Key parameters were analysed in the context of the literature to inform standardised reporting criteria.

A 36-year-old woman (G3P2) was diagnosed with an umbilical cord hemangioma at 29 + 3 weeks gestation following a routine growth scan. Serial ultrasound scans demonstrated lesion stability until 35 + 4 weeks, prompting increased fetal surveillance. Multidisciplinary consensus favoured expectant management, leading to an uncomplicated spontaneous vaginal delivery at 39 weeks. Histopathology confirmed a cord hemangioma composed of dilated, ectatic vascular channels lined by endothelial cells.

This case contributes to the growing body of evidence on umbilical cord hemangiomas by providing detailed clinical, ultrasound, and histopathological findings. Successful expectant management and favourable perinatal outcomes highlight the role of serial ultrasound surveillance. Given the rarity and potential risks of these lesions, standardised reporting is essential to improve understanding and to guide management. By proposing a set of standardised reporting criteria, this case report serves as a step toward enhancing data consistency and informing management strategies.

## Highlights

• Umbilical cord hemangiomas are rare, with no standardised reporting criteria.
• Serial ultrasound can monitor lesion stability, vessel patency, and fetal well-being.
• Conservative management is feasible if no fetal compromise is detected.
• Lesion growth may be sporadic, underscoring the need for ongoing monitoring.
• A standardised reporting framework is proposed to enhance future case documentation and management.

## 1 Introduction

Hemangiomas of the umbilical cord are rare, benign vascular anomalies characterised by the proliferation of capillary endothelial cells, typically originating from the umbilical vessels. [1,2] They appear as fusiform, hyperechogenic masses, with vascularity and associated oedematous degeneration of Wharton's jelly within free segments of the umbilical cord. [[3], [4], [5], [6]] The recorded sizes range from 2 to 180 mm in diameter. [1,4,7,8]

The literature on umbilical cord hemangiomas is sparse, comprising primarily individual case reports. A multi-institutional case series in 2024 identified a total of 56 cases published over a 12-year period. [1] The largest case series was a total of four cases. [5] Of the combined 56 cases, 59 % resulted in healthy livebirths with 13 % of cases associated with severe complications and 25 % resulting in intrauterine or neonatal demise. [1] Other reported associations include increased maternal serum alpha-fetoprotein, intrauterine growth restriction (IUGR), polyhydramnios, impaired umbilical circulation, hydrops fetalis, preterm birth, and vascular malformations. [1,3,[8], [9], [10], [11], [12], [13], [14], [15], [16]] The scarcity of data presents a challenge in understanding the clinical significance of these lesions, with little evidence available to predict perinatal outcomes or inform the management of affected pregnancies. In their discussion, O. Ferriera et al. note that more consistent documentation of pathologic features and fetal outcomes is paramount to progress understanding of these lesions. This case of umbilical cord hemangioma is presented alongside proposed standardised reporting criteria, contributing to the limited available data in this area.

## 2 Case Presentation

A 36-year-old woman, G3P2, presented at 12 + 3 weeks gestation for routine antenatal care. She had had two previous normal vaginal deliveries complicated by insulin-dependent gestational diabetes mellitus (IDGDM). IDGDM was diagnosed at 7 weeks gestation in this pregnancy. Antenatal screening was otherwise unremarkable. Investigations for blurred vision prior to this pregnancy diagnosed a 1.5 mm right posterior communicating artery (PCA) aneurysm, which was managed expectantly.

**Fig. 1:**
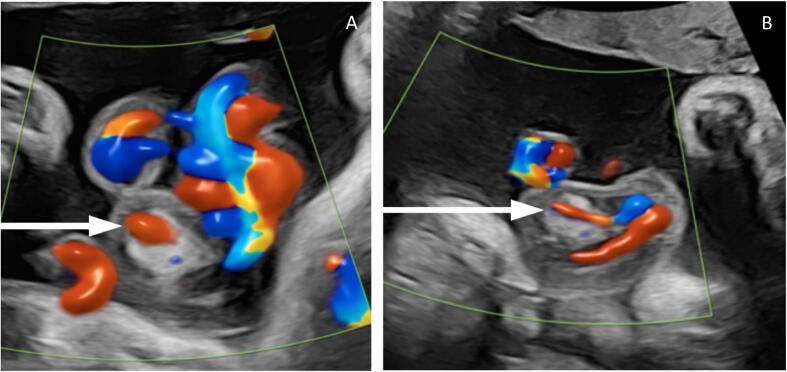
Colour Doppler ultrasound images demonstrating an umbilical cord hemangioma with a vessel seen within the lesion (indicated by arrows).

A borderline abdominal circumference measurement at the 20-week morphology scan prompted a growth ultrasound at 29 + 3 weeks, which identified an umbilical cord mass. A tertiary scan confirmed a mass within the mid-portion of the umbilical cord measuring 28 × 14 × 11 mm encasing one of the umbilical arteries with surrounding oedematous Wharton's jelly (see Fig. 1). There was no evidence of arterial pressure effect or stenosis.

**Fig. 2:**
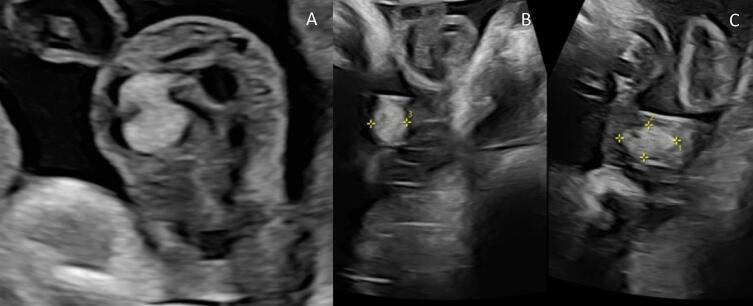
Ultrasound images demonstrating an echogenic mass within the umbilical cord, consistent with an umbilical cord hemangioma.

As there is a suggested increased likelihood of fetal growth restriction and impaired fetal circulation, serial ultrasound scans were performed. The lesion remained stable in size although an additional feeding artery was identified at 32 weeks. There was no evidence of arterial compression or alterations in flow velocity or vascular resistance. The lesion increased in size at 35 + 4 weeks to 35 × 17 × 14 mm (see Fig. 2), leading to increased fetal surveillance. No further growth of the lesion was identified. With multidisciplinary input, and in the absence of evidence supporting early delivery, an induction of labour was performed at 39 weeks with continuous electronic fetal monitoring (CEFM), which resulted in a normal vaginal delivery of a healthy female infant.

**Fig. 3:**
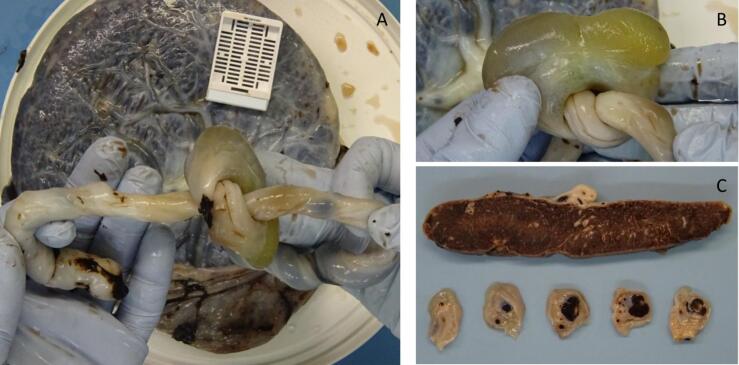
Gross examination of an umbilical cord hemangioma. (A) The lesion appears well-demarcated within Wharton's jelly, with an incidental finding of a true knot in the umbilical cord (B) A closer view highlights the fusiform shape of the mass. (C) Serial cross-sections of the lesion, and section of placental disc.

Gross examination of the umbilical cord revealed a three-vessel cord, inserted centrally, 451 mm in length, with an incidental finding of a true knot and area of cord dilation 33 mm in diameter (see Fig. 3).

**Fig. 4:**
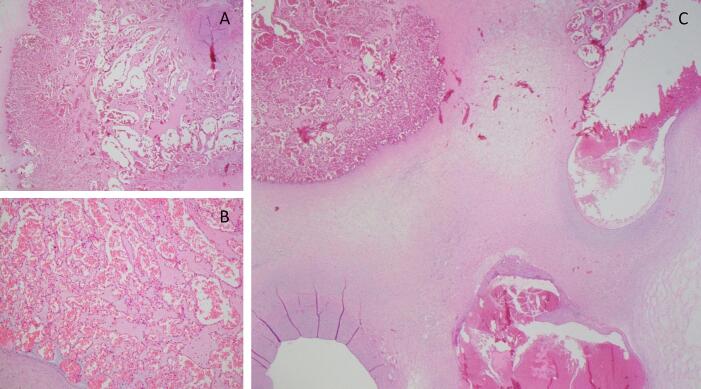
Histopathological examination of the umbilical cord hemangioma, with low-power view (C), showing the relation to umbilical vessels, and higher power (A, B) showing numerous dilated vascular channels.

Histopathological evaluation confirmed the 30 mm lesion located in the umbilical cord to be an umbilical hemangioma, characterised by numerous dilated and ectatic thin-walled vascular channels, lined by bland endothelial cells (CD31+). A few vessels in the hemangioma appeared thrombosed; however, no thrombotic vasculopathy was seen otherwise. The chorionic villi appeared well perfused and there were no features of fetal vascular malperfusion (see Fig. 4).

## 3 Discussion

Umbilical cord hemangiomas are exceedingly rare, and optimal management is complicated by the scarcity of evidence in the literature. Most information comes from isolated case reports, leaving clinicians without standardised guidelines to inform practice. In this case, serial growth scans proved valuable for monitoring the lesion throughout pregnancy, providing detailed assessment of size, stability, and potential impact on umbilical vessel patency. Growth of the lesion was not linear and appeared sporadic, suggesting that monitoring should continue throughout pregnancy. Reassuringly, there was no evidence of fetal compromise, anaemia, growth restriction, hydrops fetalis or umbilical cord compression in this case.

There has been no other reported case of fetal hemangiomas in association with a maternal PCA aneurysm or other maternal vascular or connective tissue disorders, although case reports are limited. This case raised the possibility of a potential vascular link, emphasising the need for further investigation. Notably, umbilical hemangiomas have been reported in fetal Klippel-Trenaunay-Weber syndrome and multiple cutaneous hemangiomas. [8,12,17]

The decision to proceed with conservative management was supported by stable serial assessments and the absence of evidence advocating for early delivery or mode of delivery in similar cases. However, the paucity of robust data on these rare lesions reinforces the need for further research to guide management strategies and improve outcomes in future pregnancies. Key parameters of this case have been documented in alignment with those initially collated by O. Ferriera et al. (see Table 1). Based on these parameters, a set of standardised reporting criteria is proposed to improve consistency in future case documentation. This case contributes to the growing body of evidence on the clinical outcomes and histopathological features of this extremely rare entity, and further underscores the importance of individualised care plans and the critical role of multidisciplinary collaboration in managing pregnancies complicated by umbilical cord hemangiomas.

**Table 1:** Clinical and Histopathological Characteristics of Umbilical Cord Hemangioma.

Gestational age at diagnosis	Size (mm)	Volume (cc)	Location on cord	Vessel(s) involved	Turbulent flow (Yes/No)	Cystic change (Yes/No)	Other fetal systemic vascular anomalies	Fetal outcome
29 + 3	30 mm	Not specified	170 mm from fetal insertion	Not specified	No	No	Decidual arteriopathy	Healthy
